# Electrochemical Deposition and Dissolution of Thallium from Sulfate Solutions

**DOI:** 10.1155/2015/357514

**Published:** 2015-05-03

**Authors:** Ye. Zh. Ussipbekova, G. A. Seilkhanova, Ch. Jeyabharathi, F. Scholz, A. P. Kurbatov, M. K. Nauryzbaev, A. Berezovskiy

**Affiliations:** ^1^Faculty of Chemistry and Chemical Technology, Al-Farabi Kazakh National University, 71 Al-Farabi Avenue, Almaty 050040, Kazakhstan; ^2^Institut für Biochemie, Universität Greifswald, Soldmannstrasse 23, 17489 Greifswald, Germany

## Abstract

The electrochemical behavior of thallium was studied on glassy carbon electrodes in sulfate solutions. Cyclic voltammetry was used to study the kinetics of the electrode processes and to determine the nature of the limiting step of the cathodic reduction of thallium ions. According to the dependence of current on stirring rate and scan rate, this process is diffusion limited. Chronocoulometry showed that the electrodeposition can be performed with a current efficiency of up to 96% in the absence of oxygen.

## 1. Introduction

The development of new branches of science and technology dramatically increases the demand for nonferrous metals used in various industries. Today it is difficult to find a technical field in which nonferrous metals, their alloys, and compound do not play an important role. In particular, thallium is used for production of bearing and fusible alloys, semiconductors, as a source of *β*-radiation in the radioisotope devices. It is known that alloys containing thallium have high wear resistance, inertness to acids, and fusibility [1–3]. The high toxicity and volatility of thallium compounds are not fundamental obstacles to its use [4–6]. Metals like Ga, In, and Tl are mainly distributed in the form of isomorphous impurity in minerals of other elements, with which they are extracted simultaneously and later separated. For the latter, the development of environmental friendly technologies, especially electrochemical processes, is of great importance. In order to develop a technology for the electrolytic separation and refining of thallium it is necessary to study its electrochemical behavior in different electrolytes, in order to establish the influence of the nature of electrolytes, electrolysis conditions, and so forth, on the cathodic electrodeposition and anodic dissolution of that metal. The anodic polarization curves using smooth rotating polycrystalline thallium electrode have been investigated in alkali, sulfate and acetate solutions with different ionic strengths (0.01–1.2) and pH (0.5–13.8), and using Tl_2_SO_4_ concentrations in the range of 0.1–10 mmol L^−1^ [7]. The electrochemical dissolution of thallium from the surface of a mercury film electrode has been also studied in different supporting electrolytes [8]. The authors have considered the effects of the thallium concentration, of deposition potential and deposition time and of the potential sweep rate. The deposition of thallium has also been investigated using n-Si electrodes [9]. With the help of an electrochemical quartz microbalance and voltammetry it has been shown [10] that thallium(I) ions adsorbed on a positively charged thin film mercury electrode, probably in the form of ion pairs, are not undergoing a reduction. At the potentials where adsorption was observed, only dissolved thallium(I) ions were reduced directly from the solution. Results of the study of the electrochemical behavior of thallium in a solution of 0.25 mol L^−1^ solution of hydrochloric acid on gallium and mercury electrodes are presented in [11]. Generally, the electrochemical deposition and dissolution of thallium have been insufficiently studied so far, possibly because of the toxicity of this element. The present investigation was undertaken with the goal to widen the knowledge about possible ways to refine thallium, and for this, the deposition-dissolution behavior has been studied on glassy carbon electrodes.

## 2. Experimental Part

The electrochemical behavior of thallium was studied in sulfuric acid solutions on glassy carbon electrodes. The auxiliary electrode was a platinum electrode, a silver chloride electrode (Ag/AgCl (3 M KCl) served as reference electrode (*E* = −0.222 V versus SHE). The electrochemical measurements were carried out with a computer interfaced AUTOLAB-30 (Metrohm) potentiostat-galvanostat. The thallium(I) sulfate standard solution was prepared according to the procedure described in [12]. Cyclic polarization curves were measured at various scan rates using thallium(I) concentration of 10^−3^ mol L^−1^), at different concentrations of electrolyte (1 · 10^−3^, 1 · 10^−4^, 1 · 10^−5^ mol L^−1^) and in the temperature range of 25–65°C. The electrolyte contained 0.5 mol L^−1^ sodium sulfate. Each experiment was carried out in 5–10 replicates. The obtained data was processed by mathematical statistics method [13].

For scanning electron microscopy (SEM) the instrument Quanta 3D 200i Dual System FEI (USA) equipped with an EDX detection system was used.

## 3. Results and Discussion

**Figure 1:**
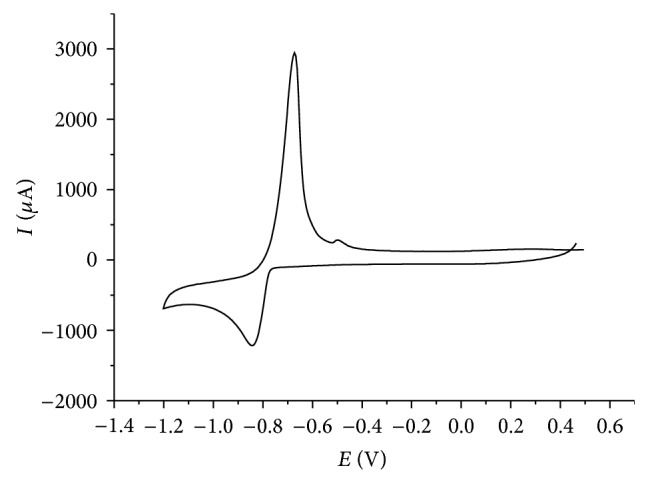
Cyclic voltammogram of Tl^+^ (*c* = 10^−3^ mol L^−1^) recorded with a glassy carbon electrode in the absence of oxygen (electrolyte 0.5 mol L^−1^ Na_2_SO_4_, pH = 7, *v* = 20 mV/s).

Figure 1 depicts a cyclic voltammogram of a 10^−3^ mol/L Tl^+^ solution in the absence of oxygen. The peak at −0.85 V corresponds to the cathodic reduction of Tl^+^ to thallium metal, and the anodic peak at −0.67 V to the anodic dissolution of the plated metal.

In order to characterize the electrochemical behavior of thallium in sodium sulfate solutions, the following parameters were varied: scan rate, temperature, and concentration of thallium sulfate.

**Figure 2:**
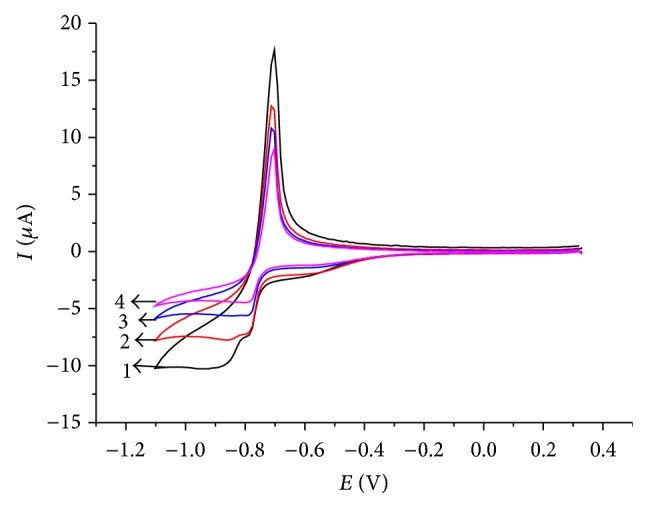
Cyclic polarization curves of Tl^+^ (*c* = 10^−3^ mol L^−1^) on glassy carbon electrode at different scan rates in the presence of oxygen (air saturated solutions). The electrolyte was 0.5 mol L^−1^ Na_2_SO_4_, pH = 7. (1) 50 mV/s; (2) 20 mV/s; (3) 10 mV/s; (4) 5 mV/s.

**Figure 3:**
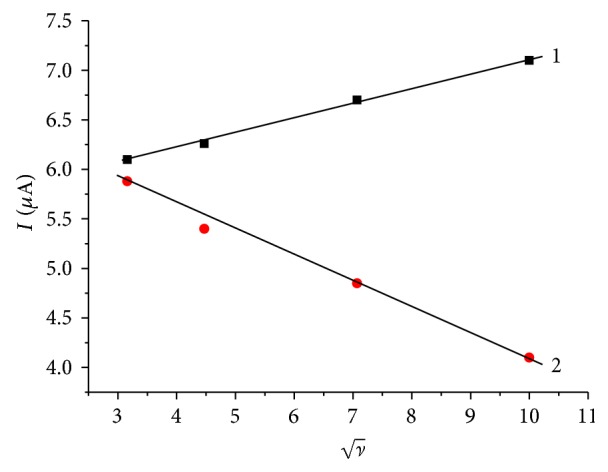
The dependence of peak currents on square root of scan rate. All experimental conditions as in Figure 2. (1) Reduction peak currents, (2) oxidation peak currents.

Unfortunately, the midpeak potential of the thallium system in the CV shown in Figure 1 is −0.74 V, which is rather negative and in the range where oxygen is reduced. If one is interested in performing an electrochemical deposition of thallium under technical conditions, the presence of oxygen should be tolerated, as any kind of deaeration is unrealistic. Therefore, it was interesting to study the electrochemical behavior in the presence of oxygen at concentrations corresponding to the partial pressure of oxygen in ambient air.  Figure 2 shows the results of experiments using different scan rates (*v*) in the presence of oxygen. The peak currents increase with increasing scan rate, exactly with the square root of scan rate (Figure 3), as it is typical for a processes controlled by semi-infinite planar diffusion. The cathodic signal at −0.4 V shows the presence of oxygen (reduction of oxygen to hydrogen peroxide). At higher scan rates, the reduction of hydrogen peroxide to water is also visible at potentials more negative than the reduction of Tl^+^.

**Figure 4:**
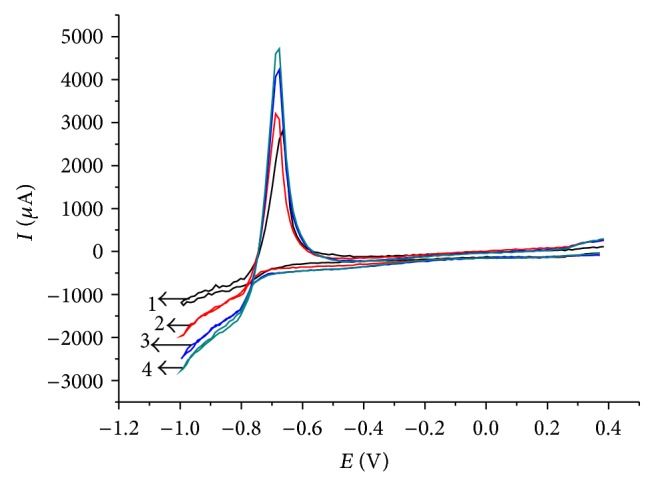
Cyclic voltammograms of Tl^+^ (*c* = 10^−3^ mol L^−1^) at a glassy carbon electrode at different stirring rates, *v* = 20 mV/s. The electrolyte was 0.5 mol L^−1^ Na_2_SO_4_, pH = 7. (1) 100 rev/min, (2) 250 rev/min, (3) 500 rev/min, and (4) 750 rev/min.

**Figure 5:**
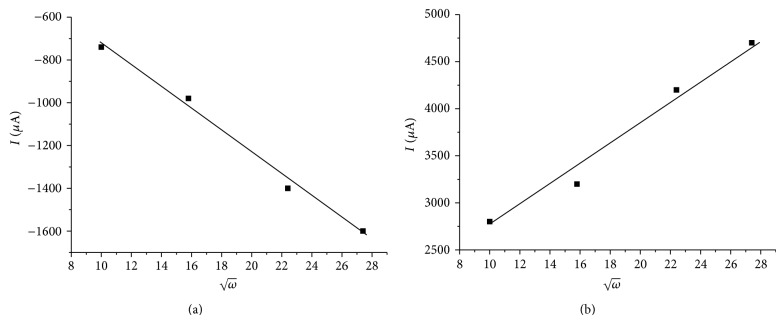
The dependence of the current density of the cathode (a) and anode (b) peaks on $\sqrt{\omega }$, where *ω* is angular frequency of the stirrer. Conditions as given in Figure 4.

Although the scan rate dependence of peak currents already indicated semi-infinite planar diffusion as the limiting process of Tl^+^ reduction, it was also attempted to test this diffusion regime by measuring the peak currents in dependence on stirring rate. Normally this is done with the help of a rotating disc electrode. Since such electrode was not available in this research, the stationary glassy carbon disc electrode was carefully placed axially above a magnetic bar for stirring the solution. The rotation rate of that bar was controlled. Although, the hydrodynamic conditions of a rotating disc electrode and of a stationary disc electrode with a stirred solution differ considerably, it was interesting to see that also in the latter a linear dependence of limiting currents on the square root of the angular frequency of the stirring bar could be observed (cf. Figures 4 and 5). This is unlikely an accidental result and can be taken as a second indication of the semi-infinite planar diffusion limitation of currents.

**Figure 6:**
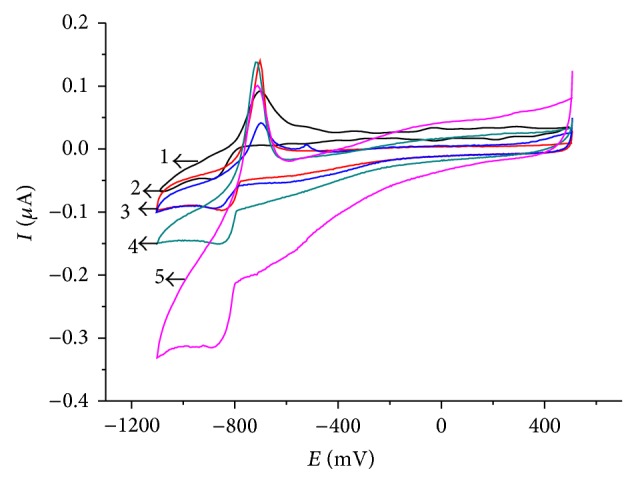
Cyclic polarization curves on the glassy carbon electrode at different temperatures in the presence of oxygen. Conditions as given in Figure 4. (1) 25°C, (2) 35°C, (3) 45°C, (4) 55°C, and (5) 65°C.

**Figure 7:**
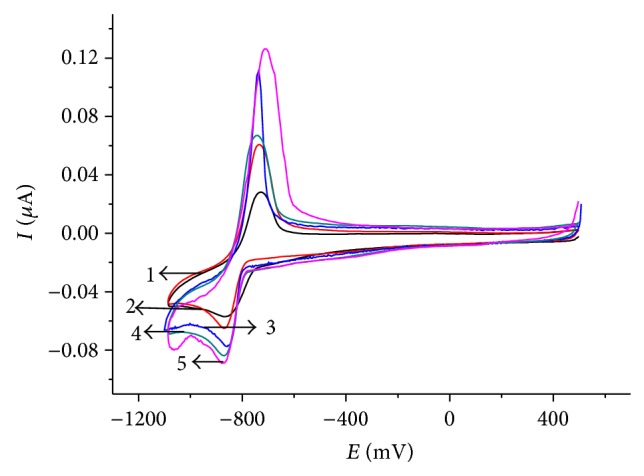
Cyclic polarization curves on the glassy carbon electrode at different temperatures in the absence of oxygen. Conditions as given in Figure 4. (1) 25°C, (2) 35°C, (3) 45°C, (4) 55°C, and (5) 65°C.

Figures 6 and 7 show the effect of temperature on the voltammetric system of thallium. Increasing temperature leads to a significant increase in reduction and oxidation currents, which is expected for diffusion limited currents. Cathodic peaks are observed at potentials of −0.85, −0.9 V and anodic at −0.78 V. In the cathode region, as seen from the curve (see Figure 6), there is another peak at the potential of −0.4 V, which, as stated earlier, corresponds to the reduction of oxygen contained in the thallium electrolyte.

**Figure 8:**
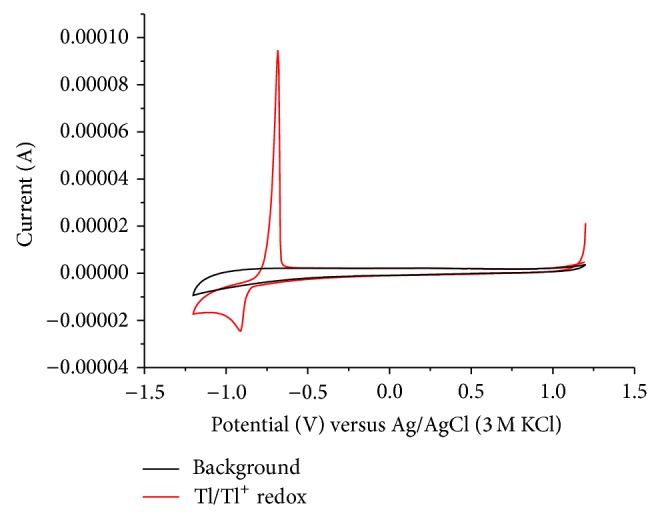
Cyclic voltammograms of glassy carbon electrode in the presence (red) and absence (black) of 1 mM TlSO_4_ in deaerated 0.5 M Na_2_SO_4_ solution.

**Figure 9:**
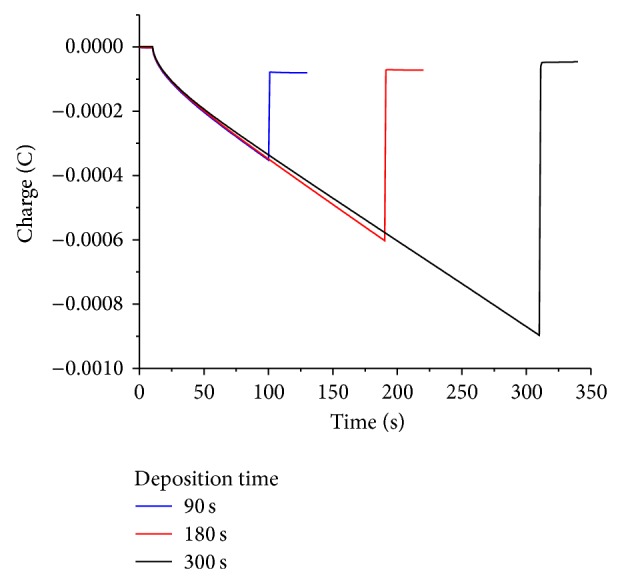
Chronocoulometry plot of thallium deposition with varying time and removal on glassy carbon electrode in the presence of 1 mM TlSO_4_ in deaerated 0.5 M Na_2_SO_4_ solution.

**Figure 10:**
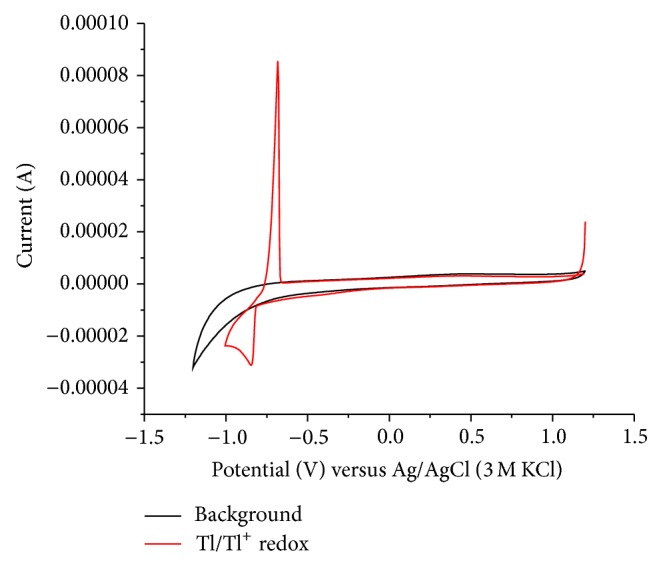
Cyclic voltammograms of glassy carbon electrode in the presence (red) and absence (black) of 1 mM TlSO_4_ in deaerated 0.5 M H_2_SO_4_ solution.

**Figure 11:**
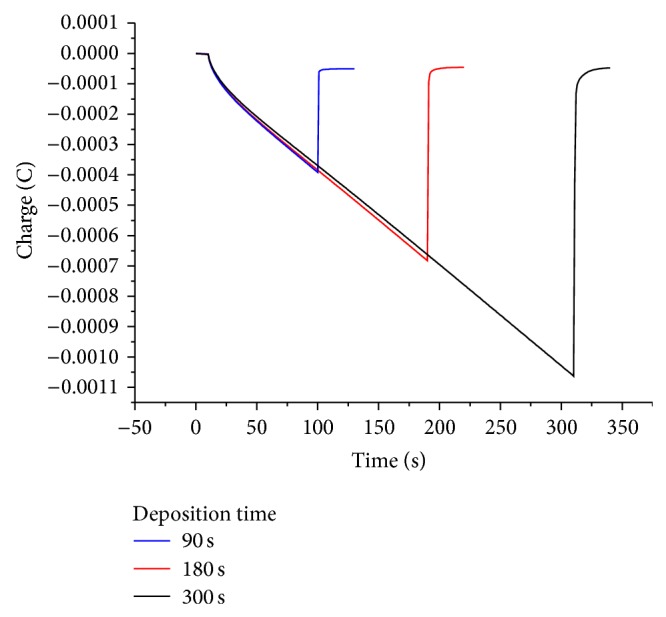
Chronocoulometry plot of thallium deposition with varying time and removal оn glassy carbon electrode in the presence of 1 mM TlSO_4_ in deaerated 0.5 M H_2_SO_4_ solution.

**Figure 12:**
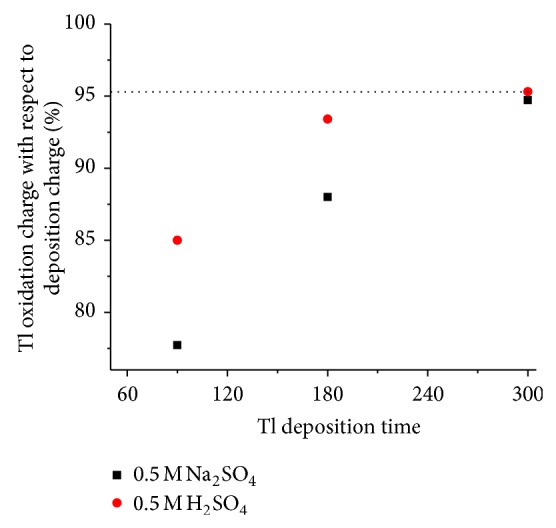
Plot shows the percentage of charge corresponding to thallium oxidation with respect to the deposition charge against its deposition time.

**Figure 13:**
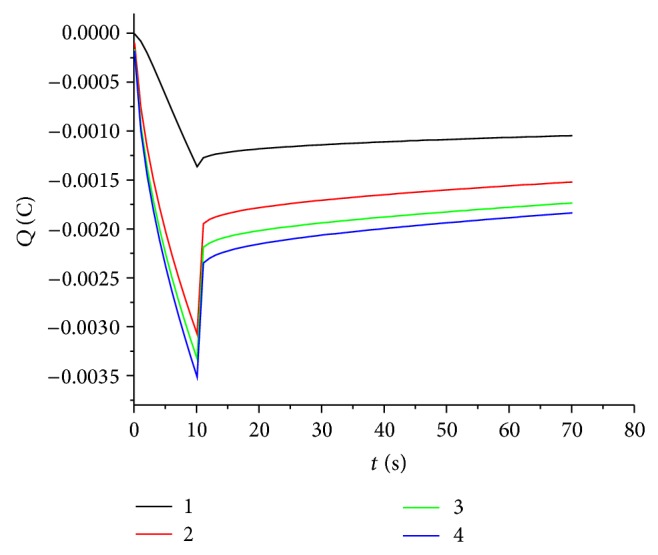
Chronocoulometry plot of thallium deposition with varying time and removal on glassy carbon electrode in the presence of 1 mM TlSO_4_ in 0.5 M Na_2_SO_4_ solution in the presence of oxygen.

Figures 8 and 10 show cyclic voltammograms in sodium sulfate and sulfuric acid solutions, respectively, and Figures 9 and 11 show chronocoulograms measured in the same electrolytes. The potential program for chronocoulometry was as follows. (i) The electrode was conditioned at −0.6 V, at which potential no reduction of Tl^+^ happens. (ii) Then a potential of −0.85 V was applied for different time intervals where Tl^+^ was reduced. (iii) Finally, a potential of −0.6 V was applied to oxidize the previously deposited thallium metal. A closer look at Figures 10 and 12 reveals a most interesting feature: independent on the deposition time, the oxidation charge never completely equals the reduction charges, but a constant charge is missing in all experiments, and this charge is the same in all experiments. This can be only understood in such way that a certain amount of deposited thallium was not oxidized under the used conditions. If that missing charge would be due to the reduction of some other impurities (e.g., traces of oxygen) the absolute amount of that charge should grow with increasing electrolysis time. Since this is not the case, it indicates that a certain amount of metallic thallium stays on the electrode without being oxidized. Using more positive potentials than −0.6 V leads to even lager differences between reduction and oxidation charges. The reasons for the differences have to be elucidated in later studies. In case of oxygen-free solutions, the highest current efficacy was 96 ± 2.5% when the reduction time was 300 s and the oxidation time was, as in all experiments, 30 s. (Figure 12). In the presence of oxygen, the efficacy was only 46 ± 1.5% for a reduction within 10 s and oxidation within 60 s (Figure 13).

From the data shown in Figure 12, one can calculate an efficiency of 46% for the deposition of Tl at different potentials. Figure 13 shows that the efficiency grows with reduction time.

**Figure 14:**
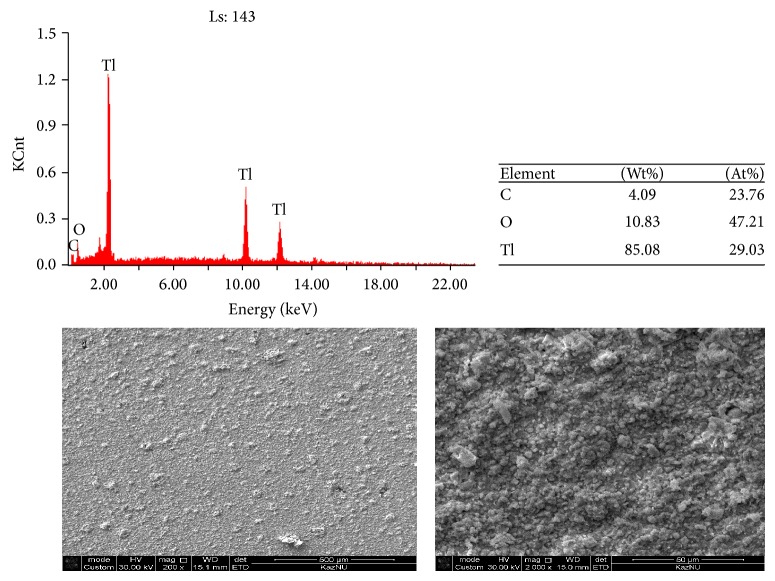
The results of the analysis of the surface of glassy carbon electrode obtained after deposition of thallium at *E* = −800 mV in 0.01 mol/L solution of Tl_2_SO_4_; background is Na_2_SO_4_, pH = 7 without stirring.

Analysis of the results of scanning electron microscopy (SEM + EDX) revealed the presence of metallic thallium at pH 7 on the surface of glassy carbon electrode after electrolysis at a constant potential corresponding to −800 mV. The surface of the electrode is covered with uniform layer of metallic thallium (Figure 14) as a silvery-white precipitate. Metallic thallium formed on the cathode metal thallium is a spongy mass, ill keep on the electrode, and is easily oxidized in air. This means that after electrolysis the precipitated thallium part falls into the solution (poor holding electrode) and the precipitate of thallium in the air is rapidly oxidized.

**Figure 15:**
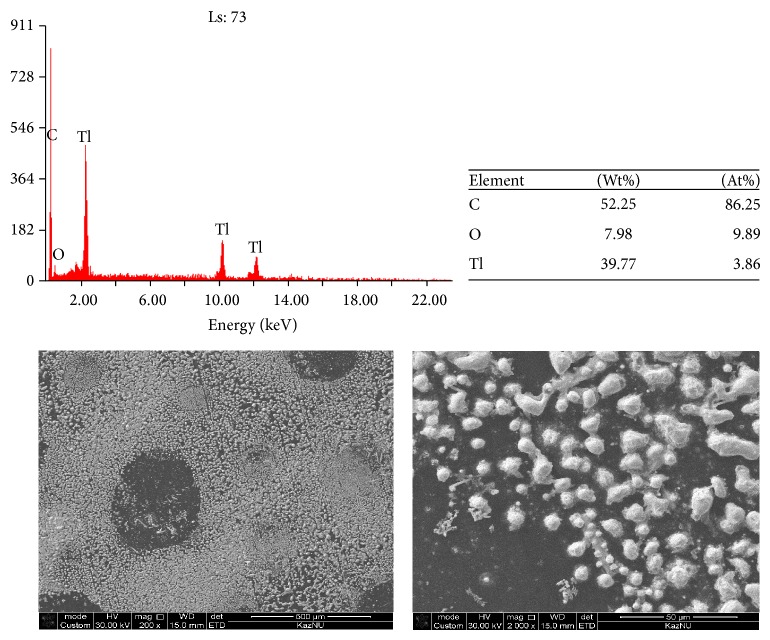
The results of the analysis of the surface of glassy carbon electrode obtained after deposition of thallium at *I* = −1300 *μ*A in 0.01 mol/L solution of Tl_2_SO_4_; background is Na_2_SO_4_, pH = 7 without stirring.

After electrolysis carried out at constant current (−1300 *μ*A), deposition of hydrogen is observed along with the process of deposition of thallium, which is confirmed by scanning electron microscopy (Figure 14). As can be seen from the figure, that is, during the deposition of thallium constant amperage (current) at the cathode with co-precipitation of thallium also released hydrogen. In this case, the precipitations of metallic thallium are of amorphous nature; deposition of hydrogen leads to the formation of uneven layer of thallium. Elemental analysis of the precipitate obtained at the constant potential showed that the content of thallium is larger than in the product precipitated at constant current (Figures 14 and 15).

Thus, the electrochemical behavior of thallium on glassy carbon electrode in sulfate solutions was studied. Linear dependence of the current density in the anode and cathode processes on the square root of the scan rate and the rate of stirring of the solution was established, indicating that the electrochemical process occurs in the diffusion mode. It was established that, based on the studies of processes of the discharge-ionization of thallium, increasing the concentration of thallium electrolyte and temperature accelerates the electrochemical reaction. The current efficiency was calculated by using the chronocoulograms measured to be 96 ± 2.5% in the absence of oxygen. The results of electron-microscopic investigation of the products of electrochemical reactions carried out at constant potential and constant current showed the formation of the metallic thallium on the surface of the glassy carbon electrode. The obtained results of the study of the electrochemical behavior of thallium may be useful in the development of the process of its refining.
